# Low Levels of Mannan-Binding Lectin or Ficolins Are Not Associated with an Increased Risk of Cytomegalovirus Disease in HIV-Infected Patients

**DOI:** 10.1371/journal.pone.0051983

**Published:** 2013-01-04

**Authors:** Adrian Egli, Juliane Schäfer, Michael Osthoff, Steffen Thiel, Christina Mikkelsen, Andri Rauch, Hans H. Hirsch, Heiner C. Bucher, James Young, Jens C. Jensenius, Manuel Battegay, Marten Trendelenburg, the Swiss HIV Cohort Study

**Affiliations:** 1 Division of Infectious Diseases and Hospital Epidemiology, University Hospital of Basel, Basel, Switzerland; 2 Clinical Immunology Laboratory, Department of Biomedicine, University of Basel, Basel, Switzerland; 3 Basel Institute for Clinical Epidemiology and Biostatistics, University Hospital of Basel, Basel, Switzerland; 4 Department of Biomedicine, University of Aarhus, Aarhus, Denmark; 5 Division of Infectious Diseases, University Hospital of Bern and University of Bern, Bern, Switzerland; 6 Department of Biomedicine, Institute for Medical Microbiology, University of Basel, Basel, Switzerland; 7 Clinic for Internal Medicine, University Hospital of Basel, Basel, Switzerland; University Medical Center Utrecht, The Netherlands

## Abstract

**Background:**

In HIV-infected patients, prediction of Cytomegalovirus (CMV) disease remains difficult. A protective role of mannan-binding lectin (MBL) and ficolins against CMV disease has been reported after transplantation, but the impact in HIV-infected patients is unclear.

**Methods:**

In a case-control study nested within the Swiss HIV Cohort Study, we investigated associations between plasma levels of MBL/ficolins and CMV disease. We compared HIV-infected patients with CMV disease (cases) to CMV-seropositive patients without CMV disease (controls) matched for CD4 T-cells, sampling time, and use of combination antiretroviral therapy. MBL and M-ficolin, L-ficolin, and H-ficolin were quantified using ELISA.

**Results:**

We analysed 105 cases and 105 matched controls. CMV disease was neither associated with MBL (odds ratio [OR] 1.03 per log_10_ ng/mL increase (95% CI 0.73–1.45)) nor with ficolins (OR per log_10_ ng/mL increase 0.66 (95% CI 0.28–1.52), 2.34 (95% CI 0.44–12.36), and 0.89 (95% CI 0.26–3.03) for M-ficolin, L-ficolin, and H-ficolin, respectively). We found no evidence of a greater association between MBL and CMV disease in patients with low CD4 counts; however in the multivariable analysis, CMV disease was more likely in patients with an increased HIV RNA (OR 1.53 per log_10_ copies/mL; 95% CI 1.08–2.16), or a shorter duration of HIV-infection (OR 0.91 per year; 95% CI 0.84–0.98).

**Conclusions:**

CMV disease is not associated with low levels of MBL/ficolins, suggesting a lack of a protective role in HIV-infected patients.

## Introduction

Cytomegalovirus (CMV) latently infects up to 70% of the general population [1]. To achieve control of CMV replication and prevent tissue-invasive CMV disease CMV-specific T-cells are essential. In patients with prolonged immunosuppression, substantial reduction of CMV-specific T-cells is associated with an increased risk for CMV reactivation, progressive replication and disease [2]. HIV-infection leads to both a significant reduction of CD4 T-cell counts and exhaustion of CD8 T-cell function. The decline of CD4 T-cells below 100 cells/µL serves as a surrogate marker for an advanced state of immunosuppression and an increased risk of CMV disease [3], [4]. Significant reductions of CMV-specific T-cells have been described in such advanced states of immunosuppression [5]. In addition, CMV disease is rare during antiretroviral treatment and after stable immune recovery. Nevertheless, in HIV-infected patients the impact of CMV-specific CD4 T-cells on CMV replication remains controversial. The increase of overall CD4 T-cell frequencies was associated with significantly less CMV disease [6]. However, Singh and colleagues reported a reduction of CMV-specific CD8 T-cells in individuals with a history of CMV disease, despite normal CD4 T-cell reconstitution [7]. In addition, CMV-specific T-cells were shown to poorly predict CMV retinitis in HIV-AIDS patients [8], suggesting a potential role for other (immunological) co-factors. In clinical states with impaired adaptive immunity such as transplantation or HIV-infection, the innate immune response may become a more decisive factor in enabling the host to gain control over virus replication.

Mannan-binding lectin (MBL) and ficolins are components of the innate immune response and are integral in complement pathway activation. MBL, an oligomer of homo-trimeric protein subunits synthesised in the liver, binds in its oligomeric form to carbohydrates on bacteria, fungi, and viruses [9], [10], [11]. MBL has been found to bind directly to virions from a number of different virus families, including human immunodeficiency virus [12], severe acute respiratory syndrome coronavirus [13], Influenza A virus [13], Ebola virus [13], dengue virus [13], and West Nile virus [13]. This interaction subsequently was shown to lead to neutralization by deposition of complement C3 and C4 on the virus subsequently preventing the interaction of the virus with the host cell and leading to increased phagocytosis or lysis of the virus [14], [15]. With regard to CMV, evidence of involvement of the lectin pathway is controversial. CMV glycoproteins are potential targets for MBL binding which could prevent virion entry into host cells. Alternatively, MBL could recognize these glycoproteins in/on the surface of an infected cell subsequently inducing complement-mediated cell destruction of the viral reservoir (infected cells) [16], [17]. In contrast, in a previous study complement activation and C3 deposition on the surface of CMV infected human skin fibroblasts were not dependent on MBL but only on C1q, the initiating molecule of the classical pathway of complement [18]. Similar to MBL, M-ficolin (ficolin-1), L-ficolin (ficolin-2), and H-ficolin (ficolin-3) also show oligomers of trimeric subunits and utilize the same activation enzymes [19], [20]. However, the effects of MBL and ficolin activation are complex and not fully understood. Intriguingly, many viruses encode homologues of complement regulatory proteins, which interact with complement receptors. These virus-induced alterations of complement function may increase virus persistence and limit immune-mediated tissue destruction [14], [21], [22]. Thus, complement may play a role in both innate and adaptive anti-viral immune responses [23].

MBL levels show only very limited acute phase changes [24], [25], and thereby a one-time measurement might be sufficient to determine life-long plasma levels. Interestingly, up to one third of the general population carry genetic polymorphisms associated with functional MBL deficiencies [26], [27]. Thus, whereas ficolin deficiencies are rare, MBL deficiencies may constitute the most common immune deficit in humans [28]. Functional deficiencies of MBL were found to be associated with an increased risk for CMV disease in recipients of solid organ and hematopoietic stem cell transplants [9], [29], [30], [31], [32], [33].

However, data on the role of functional MBL deficiency due to MBL polymorphisms in transplant patients with CMV replication are conflicting [9], [29], [30], [31], [32], [33], [34], [35]. This highlights the need to further define the impact of functional MBL deficiency in different populations with impaired immune function. The relevance of MBL and ficolins in HIV-infected patients with respect to CMV disease is unknown.

We hypothesised that functional MBL deficiency is a risk factor for CMV disease in HIV patients and therefore that HIV-infected patients with CMV disease have lower serum MBL and ficolin levels when compared to matched HIV-infected individuals with CMV antibodies but without CMV disease. Furthermore, MBL and ficolins might confer enhanced protection against CMV disease as immunodeficiency increases.

## Patients and Methods

We conducted a matched case-control study, which was nested within the Swiss HIV Cohort Study (SHCS, main centres Basel, Bern, Geneva, Lausanne, Lugano, St. Gallen, and Zürich; described in detail elsewhere [36]). We determined plasma levels of MBL (primary exposure variable) and M-ficolin (ficolin-1), L-ficolin (ficolin-2), and H-ficolin (ficolin-3) respectively (secondary exposure variables). Two groups of patients were compared: HIV-infected patients with CMV disease (cases), and HIV-infected individuals with CMV antibodies but without CMV disease (controls). Patients with other concurrent opportunistic diseases were not excluded.

### Ethics Statement

All patients gave written informed consent to the enrolment into the SHCS (http://www.shcs.ch). This study was evaluated through the scientific committee of the SHCS (project number 637). Subsampling of DNA material to detect genetic differences between CMV cases and their matched controls was envisaged; however, because genetic informed consent was introduced only in July 2001, the availability of DNA was primarily driven by year of consent. For each consenting patient, we selected a DNA sample closest to the index date (the CMV disease date).

### Selection of Cases and Controls

Eligible cases were patients with incident CMV disease (for definitions see below). Cases were required to have both a CD4 T-cell count measurement and a concurrent plasma sample taken within 90 days of the date when CMV disease was first reported (the index date). Where available CD4 T-cell counts and plasma samples collected at least 6 months before or after the index date were analysed for MBL and ficolin levels but neither was essential for inclusion in the study. Cases at the index date were classified into one of four treatment groups: no combination antiretroviral therapy (cART) use (“naïve group”), on continuous cART for less than 6 months (“new cART group”), on continuous cART for more than 6 months (“stable cART group”) or on/off cART (“unstable cART group”), where cART was defined as use of at least three antiretroviral agents. Patients on suboptimal cART (mono or dual therapy) at the index date were included in the naïve group. After an interruption to therapy for more than 6 months patients were considered to be naïve once again.

Eligible controls were positive for IgG anti-CMV, conferring a risk for CMV reactivation. Patients were not eligible as controls after starting a treatment known to have CMV anti-viral activity (val−/acyclovir, cidofovir, entecavir, famciclovir, foscarnet, val−/ganciclovir, interferon alpha, peginterferon alpha, ribavirin or telbivudine). However, they became eligible again 30 days after discontinuation of the anti-viral treatment. Controls were a random sample of all those at risk for CMV disease at the time when the index case was detected (and could include the case itself, those that were cases at a later date, or those acting as controls for other cases; [37]).

We used 1∶1 matching, i.e., we randomly sampled one control for each case. Controls were additionally matched for CD4 T-cell count (values within ±15 cells/µL of the index case), and for cART treatment classification as one of the four groups (see above). We used matching to ensure adjustment for CD4 T-cell count and cART classification and to allow us to consider homogeneity of the association between CMV disease and MBL across CD4 T-cell count strata. As with cases, where available control CD4 T-cell counts and plasma samples collected at least 6 months before or after the index date were analysed for MBL and ficolin levels.

### Definitions and Endpoints

Infectious outcomes were classified according to previously published criteria ([2] and http://www.shcs.ch/56-definitions#4.5.3). “CMV disease” included CMV retinitis and other end-organ involvement excluding liver, spleen and lymph node disease. CMV infections of the liver, lymph nodes and spleen were not considered as these rare diseases are excluded in the CDC AIDS defining conditions (http://www.cdc.gov/mmwr/preview/mmwrhtml/rr5710a2.htm). Case definitions were according to international and standardized criteria and this guarantees a high level of diagnostic accuracy. Definitive CMV retinitis was diagnosed by characteristic ophthalmological appearance, CMV-detection by polymerase chain reaction (PCR), and response to CMV treatment. However, in older cases (>10 years) the code “definitive” CMV retinitis is related to previous CDC-definitions that did not require PCR. Presumptive CMV retinitis was diagnosed by characteristic appearance on ophthalmoscopic examination (discrete patches of retinal whitening with distinct borders spreading in a centrifugal manner, frequently associated with retinal vasculitis, haemorrhage and necrosis) and typical findings following the resolution of active disease (residual retinal scarring and atrophy with retinal pigment epithelial mottling). Invasive CMV disease at other sites was always definitive with histologically proven CMV-infection. A positive culture alone from any site (except cerebrospinal fluid) without histological evidence of invasive disease was not considered sufficient for the diagnosis of CMV disease.

### Determination of MBL and M- (1), L- (2) and H-ficolin (3) Levels and MBL Genotyping

For each parameter, all samples were thawed and analysed simultaneously. MBL, and L-ficolin, H-ficolin levels were quantified using commercially available ELISA kits (MBL: Bioporto®, Denmark; H- and L-ficolin: Hycult Biotechnology®, Netherlands). All assays were performed following the manufacturer’s instructions. Levels of M-ficolin were determined using an in-house time-resolved immunofluorometric assay developed by Wittenborn et al. as previously published [38]. In brief, wells were coated with monoclonal anti-M-ficolin, incubated with samples diluted in a high ionic strength buffer with EDTA, heat aggregated human IgG and bovine IgG, washed and developed with biotinylated anti-M-ficolin antibody followed by europium-labelled streptavidin, and read on a time-resolved fluorometer.

MBL genotyping was performed as previously described [39].

### Power Calculations

Conditional logistic regression is appropriate to assess the association between CMV disease and plasma MBL in a study with matched pairs [40]. Assuming a common standard deviation regarding MBL among matched sets and a 1-sided type I error rate of 2.5%, with 126 cases and their 126 matched controls a power of 80% could be achieved to detect an odds ratio for CMV disease of 1.4 per standard deviation unit decrease in MBL [41].

### Statistical Analyses

We used conditional logistic regression to estimate associations between CMV disease and plasma MBL/M- (1), L- (2), or H-ficolin (3). In univariable analyses, we estimated the associations between CMV disease and the four individual proteins, unadjusted for covariates other than the matching factors. In the multivariable analysis, we assessed the association between CMV disease and MBL adjusted for additional potentially confounding risk factors for CMV disease, namely age, gender, HIV RNA and duration of HIV-infection. For patients without a date of a first documented or reported positive test, we used the date of the SHCS cohort registration visit to calculate an approximate duration of HIV-infection.

A key question for this study was whether MBL confers enhanced protection against CMV disease as immunodeficiency increases. To address this question, we added a covariate for the interaction between CD4 T-cell count and MBL to our model. The estimate for this interaction shows how the association between CMV disease and MBL changes at lower CD4 T-cell counts. In addition, we estimated the association between CMV disease and MBL depending on CD4 T-cell count. We calculated CD4 T-cell count strata to separate patients with CD4 T-cell counts below 20 cells/µL from those with CD4 T-cell counts above 20 cells/µL.

Earlier studies, which used MBL in categories rather than on a continuous scale, have suggested a protective role of MBL against CMV disease in transplant recipients [32]. In an additional analysis, we therefore represented MBL by a piecewise linear spline with 2 knots at 100 and 500 ng/mL. Using a piecewise linear spline allows estimation of the association between CMV disease and MBL without assuming a continuous gradient across the entire range of observed MBL levels, but rather below 100, in between 100 and 500 and above 500 ng/mL.

With plasma samples obtained at least 6 months before or after the date when CMV disease was first diagnosed not being essential for patient eligibility, we refrained from formal analyses in order to avoid a possible selection bias but rather plotted profiles from past measurements through index date measurements and measurements after the index date. Sub-cohort genetic data were summarised using descriptive statistics.

For each analysis, we report an estimate of the odds ratio with 95% confidence interval assessing the association between CMV disease and MBL/ficolin levels. We used SAS version 9.2 (SAS Institute Inc., Cary, NC, USA) for our analyses; and for graphics, we used R version 2.14.2 (R Foundation for Statistical Computing, Vienna, Austria) and the R add-on package *lattice* version 0.20–6 [42].

## Results

### Patient Characteristics

As of May 2010, 779 cases of incident CMV disease were documented in the Swiss HIV Cohort Study (SHCS). Of these, 126 (16%) patients had both a CD4 T-cell count measurement within ±90 days of the date of CMV disease diagnosis, in addition to a concurrently drawn plasma sample. Ultimately, plasma samples could be retrieved for 105 (13%) cases, with a first episode of CMV disease between January 1995 and October 2008, and for their 105 matched controls. These patients were included in the study and corresponding plasma samples analysed for MBL and ficolin levels. Excluded cases, with a first episode of CMV disease between March 1985 and November 2009, had on average a shorter duration of HIV-infection, and were more likely to be male with no prior exposure to cART (**Table 1**).

**Table 1:** Patient characteristics for included and excluded cytomegalovirus (CMV) disease cases.

Characteristic	Measure or category	Included cases	Excluded cases
		(n = 105)	(n = 674)
Age, years	Median	37	37
	(IQR)	(32–44)	(32–45)
Gender, %	Male	70	82
	Female	30	18
CMV disease^1^, %	Not liver, spleen or lymph nodes	49	43
	Retinitis	51	57
Stage of infection^2^, %	CDC group A	13	11
	CDC group B	11	12
	CDC group C	75	77
Duration of HIV-infection^3^, years	Median	4.9	3.0
	(IQR)	(0.5–8.1)	(1.3–6.1)
Other concurrent opportunistic infections^4^, %	Yes	11	16
	No	89	84
History of cART use, %	Naïve	59	92
	New cART	18	5
	Stable cART	6	1
	Unstable cART	17	3

*Abbreviations:* IQR, interquartile range; CMV, cytomegalovirus; HIV, human immunodeficiency virus; cART, combination antiretroviral therapy; IRIS, immune reconstitution inflammatory syndrome.

1 Cases diagnosed with both “CMV not liver, spleen or lymph nodes” and “CMV retinitis” are counted as the former (n = 2 and n = 20 for included and excluded CMV disease cases respectively).

2 These statistics represent the clinical stage a patient had prior to the index date.

3 Based on the date of (for included and excluded CMV disease cases respectively): a first documented positive test (49%, 39%), a first reported positive test (33%, 38%) or the cohort registration visit (18%, 22%).

4 Herpes simplex disease, mucocutan. Herpes simplex ulceration, herpes zoster multidermatomal or relapse, disseminated toxoplasmosis, toxoplasmosis retinitis or toxoplasmosis of the brain within ±90 days of the index date.

Patient characteristics for included cases and their controls are summarised in **Table 2**. Cases were predominantly male with a median age of 37 years (interquartile range [IQR] 32–44). CMV retinitis (51%) was the most important location among cases. Two patients with CMV retinitis had concurrent involvement of other end-organ sites. Fifteen patients diagnosed with CMV retinitis had a presumptive diagnosis only, one of whom also had simultaneous extra-retinal disease. More than half of the patients had no history of prior exposure to cART; median CD4 T-cell count was 20 cells/µL. According to the CDC classification system for HIV-infected patients, 75% of the cases were classified as having CDC stage C. Cases had on average a shorter duration of HIV-infection and lower CD8 T-cell count compared with their controls, but higher HIV RNA levels.

**Table 2:** Patient characteristics for included cases and their controls.

Characteristic	Measure or category	Cases	Controls
		(n = 105)	(n = 105)
Age, years	Median	37	37
	(IQR)	(32–44)	(33–42)
Gender, %	Male	70	74
	Female	30	26
CMV disease^1^, %	Not liver, spleen or lymph nodes	49	
	Retinitis	51	
Stage of infection^2^, %	CDC group A	13	11
	CDC group B	11	31
	CDC group C	75	57
Duration of HIV-infection^3^, years	Median	4.9	5.5
	(IQR)	(0.5–8.1)	(2.3–10.1)
HIV RNA^4^, log_10_ copies/mL	Median	5.0	4.4
	(IQR)	(4.1–5.4)	(3.0–5.2)
CD4 T-cell count^5^, cells/mm^3^	Median	20	22
	(IQR)	(10–61)	(10–65)
CD8 T-cell count, cells/mm^3^	Median	396	516
	(IQR)	(214–648)	(290–838)
Other concurrent opportunistic infections^6^, %	Yes	11	5
	No	89	95
History of cART use^5^, %	Naïve	59	59
	New cART	18	18
	Stable cART	6	6
	Unstable cART	17	17
MBL, log_10_ ng/mL	Median	3.2	3.1
	(IQR)	(2.6–3.4)	(2.6–3.5)
H-ficolin, log_10_ ng/mL	Median	4.6	4.6
	(IQR)	(4.4–4.8)	(4.3–4.8)
L-ficolin, log_10_ ng/mL	Median	4.3	4.3
	(IQR)	(4.2–4.4)	(4.2–4.4)
M-ficolin, log_10_ ng/mL	Median	3.0	3.1
	(IQR)	(2.9–3.2)	(2.9–3.3)
MBL structural genotype^7^, %	A/A	52	56
	A/O	48	40
	O/O	0	4

*Abbreviations:* IQR, interquartile range; CMV, cytomegalovirus; HIV, human immunodeficiency virus; cART, combination antiretroviral therapy; IRIS, immune reconstitution inflammatory syndrome; MBL, mannan-binding lectin.

1 Cases diagnosed with both “CMV not liver, spleen or lymph nodes” and “CMV retinitis” are counted as the former (n = 2).

2 These statistics represent the clinical stage a patient had prior to the index date.

3 Based on the date of (for included cases and their controls respectively): a first documented positive test (49%, 52%) a first reported positive test (33%, 35%) or the cohort registration visit (18%, 12%).

4 Available in 89 case-control pairs.

5 Matching variable.

6 Herpes simplex disease, mucocutan. Herpes simplex ulceration, herpes zoster multidermatomal or relapse, disseminated toxoplasmosis, toxoplasmosis retinitis or toxoplasmosis of the brain within ±90 days of the index date.

7 Available in 25 case-control pairs.

### MBL and Ficolin Levels in Patients with and without CMV Disease

MBL levels appeared stable over the range of age of stored samples (in years) for both cases and controls, giving no cause for concern about the quality and stability of stored plasma samples (data not shown). We used September 1^st^ 2010 to calculate an approximate age of plasma samples, although plates were tested over a time span of two weeks and a second set was tested in October 2010.

**Figure 1:**
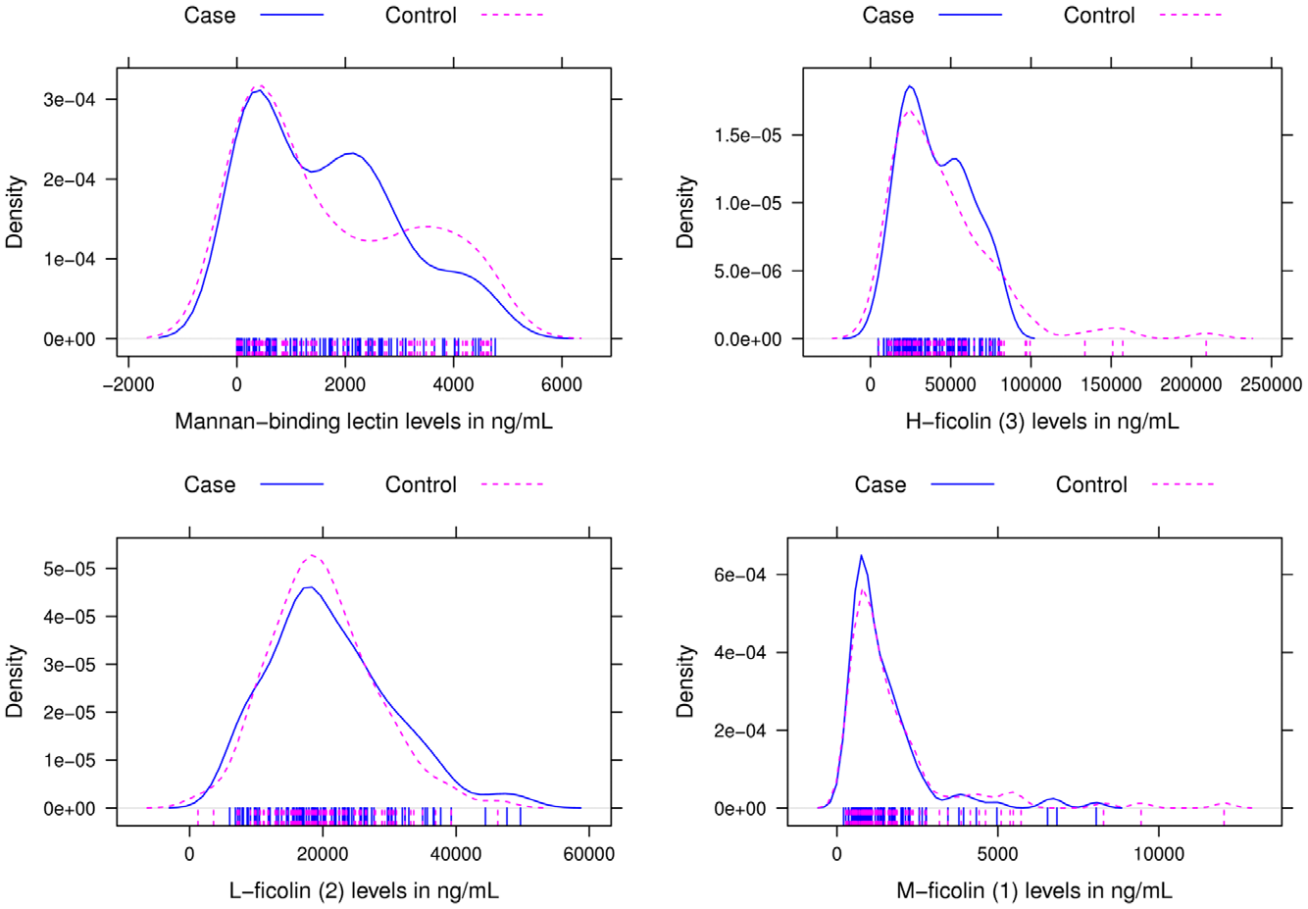
Density plots of the distribution of mannan-binding lectin (MBL), H-ficolin (3), L-ficolin (2), and M-ficolin (1) levels among cases (solid line) and controls (dashed line), with marker levels given in ng/mL.

At the time of first diagnosis of CMV disease the median MBL level for cases was 1,535 ng/mL (IQR 357–2,465). The median M-ficolin, L-ficolin, and H-ficolin levels were 1,008 ng/mL (IQR 723–1,722), 19,248 ng/mL (IQR 14,835–26,232) and 36,546 ng/mL (IQR 22,880–57,054) respectively. Controls had a median MBL level of 1,228 ng/mL (IQR 370–3,196), with median M-ficolin, L-ficolin and H-ficolin levels of 1,183 ng/mL (IQR 797–1,872), 19,068 ng/ml (IQR 15,201–24,784) and 37,391 ng/mL (IQR 21,595–57,884) respectively. Density plots of MBL and M-ficolin, L-ficolin and H-ficolin levels showed comparable distributions among cases and controls (**Figure 1**).

In univariable conditional logistic regression, no associations were found between CMV disease and any one of MBL, M-ficolin, L-ficolin, or H-ficolin (**Table 3a**). Similarly, having adjusted for potentially confounding risk factors for CMV disease other than those considered as matching variables, no association was found between CMV disease and MBL (**Table 3b**). The multivariable analysis was based on 89 case-control pairs without missing data regarding any of the considered potentially confounding risk factors for CMV disease (age, gender, HIV RNA level and duration of HIV-infection). CMV disease was more likely in females (OR 2.13; 95% CI 0.84–5.41), and with increasing HIV RNA level (OR 1.53 per log_10_ copies/mL; 95% CI 1.08–2.16), but less likely with increasing duration of HIV-infection (OR 0.91 per year; 95% CI 0.84–0.98).

**Table 3:** Associations between cytomegalovirus (CMV) disease and mannan-binding lectin (MBL)/ficolin levels.

Effect	Odds ratio	95% CI
**A**		
MBL, log_10_ ng/mL	1.03	0.73–1.45
H-ficolin, log_10_ ng/mL	0.89	0.26–3.03
L-ficolin, log_10_ ng/mL	2.34	0.44–12.36
M-ficolin, log_10_ ng/mL	0.66	0.28–1.52
**B**		
MBL, log_10_ ng/mL	0.85	0.55–1.30
Age^1^, years	1.03	0.67–1.59
Female gender	2.13	0.84–5.41
HIV RNA, log_10_ copies/mL	1.53	1.08–2.16
Duration of HIV-infection, years	0.91	0.84–0.98
**C**		
Effect of MBL per log_10_ ng/mL		
CD4 T-cell count ≤20 cells/mm^3^	0.92	0.60–1.41
CD4 T-cell count >20 cells/mm^3^	1.26	0.69–2.28
**D**		
Effect of MBL per 100 ng/mL		
≤100 ng/mL	0.71	0.12–4.15
100–500 ng/mL	1.17	0.83–1.65
>500 ng/mL	0.99	0.96–1.01

*Abbreviations:* MBL, mannan-binding lectin; CI, confidence interval.

1 Per 10 years increase.

A) Univariable conditional logistic regression models for MBL, H-ficolin (3), L-ficolin (2) and M-ficolin (1). B) Multivariable conditional logistic regression model for MBL adjusted for the effects of potentially confounding risk factors for CMV disease based on 89 case-control pairs without missing data. C) Conditional logistic regression model for MBL, investigating its relationship with CD4 T-cell count. D) Conditional logistic regression model with a linear spline for MBL.

In the present analysis, there was no evidence that MBL is a more important immunological risk factor for CMV disease in patients with CD4 T-cell counts below 20 cells/µL (OR for interaction 0.74; 95% CI 0.35–1.53). The estimated ORs were 1.26 per log_10_ ng/mL (95% CI 0.69–2.28) and 0.92 per log_10_ ng/mL (95% CI 0.60–1.41) for patients with CD4 T-cell counts above and below 20 cells/µL respectively (**Table 3c**). With the linear spline model representing the functional relationship between CMV disease and MBL by three different (below 100, in between 100 and 500, and above 500 ng/mL) as opposed to one single continuous gradient, no association was found between CMV disease and MBL either (**Table 3d**).

**Figure 2:**
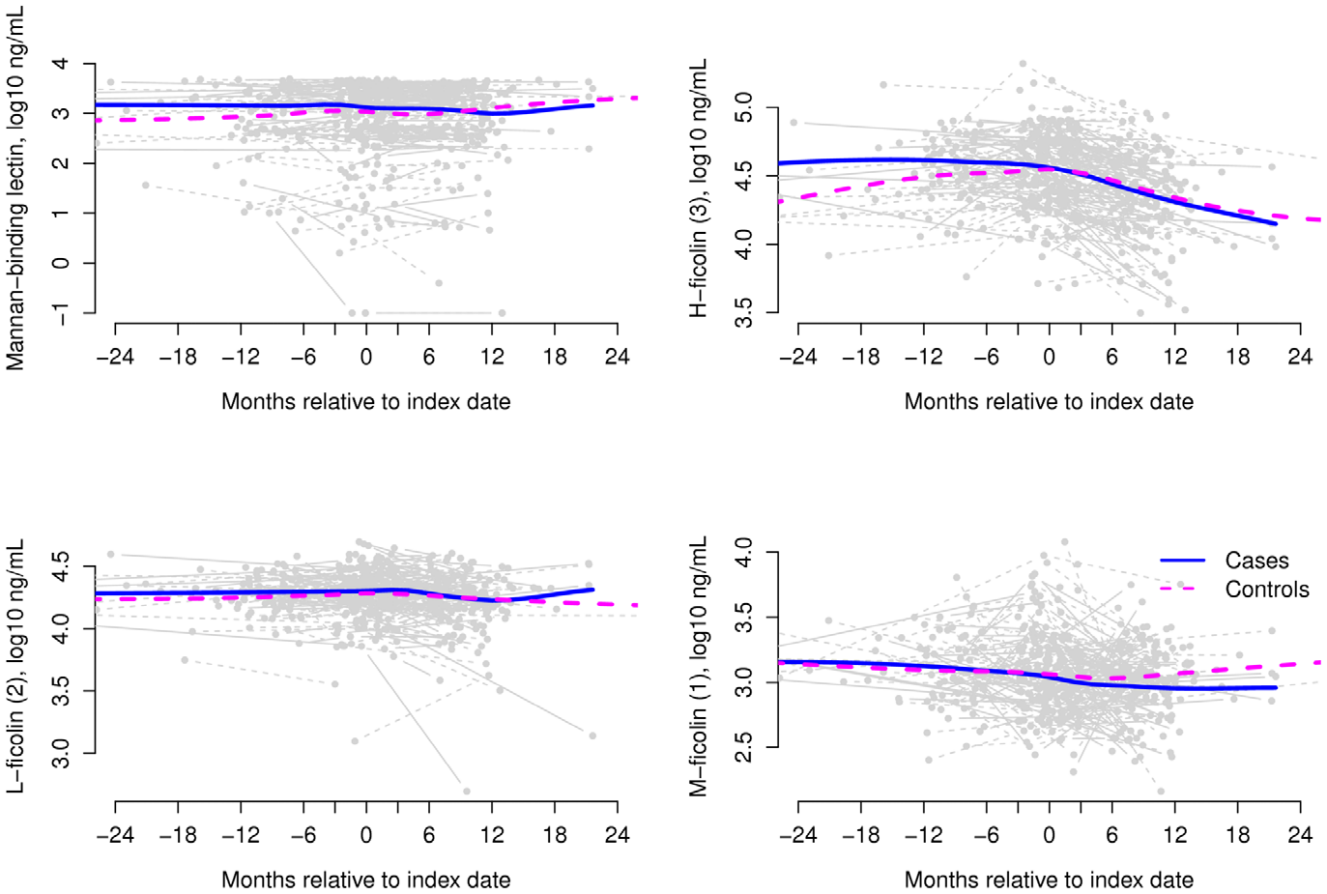
Mannan-binding lectin (MBL), H-ficolin (3), L-ficolin (2) and M-ficolin (1) levels over time for 105 case-control pairs included in this study. Time is relative to the index date, with pre and post disease measurements being optional; marker levels are given in ng/mL on a log_10_ scale. The smooth curves are local averages found using the default loess smoother in R (R Foundation for Statistical Computing, Vienna, Austria).

Plasma levels of MBL and all three ficolins appeared stable over the range of past (available in 34 (32%) cases and 50 (48%) controls) through at and after (available in 68 (65%) cases and 80 (76%) controls) disease date measurements in both cases and their matched controls (**Figure 2**). MBL levels did not appear to follow a typical pattern of acute-phase response with up-regulation during CMV disease. Prior to the index date, no phase of reduced MBL or ficolin levels was observed.

Structural MBL genotypes were available for 25 matched pairs, with similar distribution among cases and controls (**Table 2**).

We carried out a supplementary analysis to compare cases and controls with regard to MBL/ficolins by type of CMV disease. The median MBL level (log_10_ ng/ml) for cases of CMV retinitis (n = 54) and their controls was 3.2 (IQR 2.5–3.4) and 3.1 (2.7–3.6), respectively. Cases of other end-organ CMV disease (not liver, spleen or lymph nodes; n = 51) and their controls had a median MBL level (log_10_ ng/ml) of 3.2 (IQR 2.6–3.4) and 3.1 (IQR 2.4–3.4), respectively. A similar pattern of no obvious differences was seen for H-, L-, and M-ficolin.

## Discussion

To the best of our knowledge this is the first study on the potential link between MBL and CMV disease in HIV-infected patients. In a nested case-control study we compared 105 HIV-infected patients with histologically proven tissue-invasive CMV disease to a well-matched control group at risk of CMV reactivation (positive CMV-IgG, low CD4 T-cell count). Our study shows that low MBL and serum ficolin levels are not associated with an increased risk for CMV disease in HIV-infected patients. Our results further suggest that the association between MBL and CMV disease does not depend on CD4 T-cell count. However, CMV disease was more prevalent with increasing HIV RNA level and, surprisingly, less prevalent with increasing time since HIV-infection.

A major strength of this study is the large number and high quality of samples available from the multi-center SHCS. Patient samples and clinical information were prospectively collected through dedicated teams of infectious diseases specialists and HIV study nurses. In the SHCS, the diagnostic criteria for CMV disease, either CMV retinitis or CMV disease at other sites, require detection by PCR and/or histological proof of tissue-invasive disease. Therefore a high level of diagnostic accuracy is guaranteed. Our study is tightly matched with respect to immunological confounders. CD4 T-cell counts differed only by a maximum of 15 cells/µL, and cART utilisation was matched leading to a well-matched composite state of immunosuppression in cases and controls. The matching criteria allowed us to control for potential confounders of the adaptive immune response.

While we could not identify an association between low plasma MBL levels and CMV disease in HIV-infected individuals, the impact of MBL deficiency as a risk factor for CMV disease in immunosuppressed individuals remains controversial. In children with presumed primary CMV-infection, genotypes associated with lower MBL levels were more frequently observed in patients with active replication [43]. Another study failed to find a correlation between CMV replication in newborns and the frequencies of MBL genotypes [44]. After transplantation several studies have highlighted MBL deficiency as a potential risk factor for CMV replication and disease [29], [31], [32], [45], [46]. *Manuel et al.* reported an increased risk of CMV disease in a small study of high-risk CMV sero-negative recipients of CMV sero-positive kidney grafts with functional MBL deficiency [31]. The presence of MBL and ficolin-2 polymorphisms in the liver and lung transplant recipients were associated with increased CMV replication in the recipient [29], [45], [46]. However, in other transplant cohorts, either structural haplotype AA had higher rates of CMV disease compared to patients with A/O or O/O [34]. In addition, MBL and MASP-2 measurements were similar in patients with and without post-transplant CMV replication [35]. None of the studies discussed effects of immunosuppression as a potential confounder. In addition, studies showed higher proportions of functional MBL deficiency in transplantation compared to healthy individuals [47], [48], implying selection biases.

Ficolin deficiencies have been associated with an increased risk for bacterial infection. *Munthe-Fog et al.* described a case of a homozygous H-ficolin deficient patient with no detectable ficolin level and recurrent infections, suggesting a role for ficolin in the control of bacterial infection. In our study only one patient showed an H-ficolin level below 100 ng/mL. A functional protective threshold for ficolins has not yet been determined. Although we have not powered our study to determine a predictive threshold for CMV disease, our data does not indicate an association between M-ficolin, L-ficolin, and H-ficolin levels and CMV disease.

Our study presents some limitations. Patients with concurrent opportunistic infections other than CMV disease were not excluded from the control group. With regard to the occurrence of opportunistic infections in cases versus controls we could not observe an apparent dysbalance between both patient groups. In HIV-infected patients, CMV retinitis is the most common manifestation of CMV disease, whereas this disease entity is extremely rare in transplant recipients [49]. A large increase in vitreous MBL levels in patients with endophthalmitis and retinal vasculitis has been described compared to non-inflamed eyes [50]. Although speculative, this suggests that MBL levels in general might be lower in the eye and therefore biologically not active against CMV. Our study was not designed to assess differences in MBL and ficolin levels between cases with CMV retinitis and those with other disease localisation. Another limitation is the lack of information on CMV viremia and CMV replication dynamics in the context of MBL levels. Indeed, patients with high CMV viremia do show an increased risk for CMV retinitis and might benefit from a pre-emptive antiviral therapy [51]. CMV disease, particularly CMV retinitis and CMV colitis, is often associated with absent or low level CMV viremia/antigenemia. However, other studies reported a poor positive and negative predictive value of CMV viremia in regard of CMV disease [52], [53], [54]. In this study, we focused on “CMV disease” as a clinical important outcome. A prospective study might address the association between viremia and MBL in more details.

CMV-specific T-cells are known to prevent progression of CMV replication to CMV disease in immunosuppressed hosts [55]. We have not measured CMV-specific CD4+ and CD8+ T-cell responses, however this important co-factor should be included in future prospective studies.

In summary, our results suggest that low plasma MBL and ficolin levels are not associated with an increased risk of CMV disease in patients with HIV.
